# A Nanoparticle-Based Biosensor Combined With Multiple Cross Displacement Amplification for the Rapid and Visual Diagnosis of *Neisseria gonorrhoeae* in Clinical Application

**DOI:** 10.3389/fmicb.2021.747140

**Published:** 2021-10-14

**Authors:** Xu Chen, Liming Huang, Qingxue Zhou, Yan Tan, Xuhong Tan, Shilei Dong

**Affiliations:** ^1^The Second Clinical College, Guizhou University of Traditional Chinese Medicine, Guiyang, China; ^2^Clinical Medical Laboratory of the Second Affiliated Hospital, Guizhou University of Traditional Chinese Medicine, Guiyang, China; ^3^Guizhou Provincial Center for Disease Control and Prevention, Guiyang, China; ^4^Clinical Laboratory, Hangzhou Women’s Hospital, Hangzhou, China; ^5^Guizhou Provincial Center for Clinical Laboratory, Guiyang, China; ^6^Department of Clinical Laboratory, Zhejiang Hospital, Hangzhou, China

**Keywords:** gonorrhea, *Neisseria gonorrhoeae*, multiple cross displacement amplification, gold nanoparticle-based lateral flow biosensor, limit of detection, point-of-care testing

## Abstract

Gonorrhea is a sexually transmitted disease caused by the host-adapted human pathogen, *Neisseria gonorrhoeae*. The morbidity is increasing and poses a major public health concern, especially in resource-scarce regions. Therefore, a rapid, visual, sensitive, specific, cost-saving, and simple assay for *N. gonorrhoeae* detection is critical for prompt treatment and the prevention of further transmission. Here, for the first time, we report a novel assay called the multiple cross displacement amplification combined with gold nanoparticle-based lateral flow biosensor (MCDA-LFB), which we constructed for the rapid and visual identification of *N. gonorrhoeae* in clinical samples. We successfully devised a set of MCDA primers based on the *N. gonorrhoeae*-specific gene, *orf1*. Optimal assay conditions were determined at 67°C, including genomic DNA preparation (∼15 min), MCDA amplification (30 min), and LFB reading (∼2 min), which can be completed within 50 min. The limit of detection (LoD) of the assay was 20 copies/test (in a 25-μl reaction mixture). Assay specificity was 100%, with no cross-reactions with other pathogens. Thus, our *N. gonorrhoeae*-MCDA-LFB is a rapid, specific, visual, cost-saving, and easy-to-use assay for *N. gonorrhoeae* diagnostics, and may have great potential for point-of-care (POC) testing in clinical settings, especially in resource-limited regions.

## Introduction

Gonorrhea is a sexually transmitted disease caused by the host-adapted human pathogen, *Neisseria gonorrhoeae*. According to the World Health Organization (WHO), approximately 87 million new cases occur globally, of these, more than 90% occur in underdeveloped regions (Rowley et al., 2019). With an increasing global morbidity, gonorrhea poses a major public health concern (Hook and Bernstein, 2019; Quillin and Seifert, 2018). *N. gonorrhoeae* easily infects the mucosa of exposed anatomic regions, including the urogenital tract, pharynx, conjunctivae, and rectum (Chan et al., 2016; Kirkcaldy et al., 2019), and causes serious disease if not treated in a timely and appropriate manner. In females, infection leads to pelvic inflammatory disease, ectopic pregnancy, chronic pelvic pain, and tubal factor infertility. Also, infections during pregnancy may lead to the premature rupture of membranes, spontaneous abortion, preterm birth, and low birth weight (Stevens and Criss, 2018; Hook and Bernstein, 2019; Młynarczyk-Bonikowska et al., 2020). Maternal transmission to infants during birth may also lead to hyper-acute conjunctivitis, corneal perforation, and blindness (Stevens and Criss, 2018). Gonococcus infection in males can cause prostatitis and epididymo-orchitis (Abraha et al., 2018; Jefferson et al., 2021). Gonorrhea is also related to an increased risk of human immunodeficiency virus (HIV) infection and transmission (Jefferson et al., 2021). Hence, the development of an assay for *N. gonorrhoeae* diagnosis is critical for prompt treatment and the prevention of further transmission.

Traditional laboratory-based gold standard gonococcus diagnostic methods were based on cultivation. However, sensitivity was low and was largely due to poor sample collection, transport, and storage (Meyer and Buder, 2020). In addition, cultivation was time consuming and labor intensive. Nowadays, nucleic acid amplification tests (NAATs), including polymerase chain reaction (PCR) and real-time-PCR, are considered new diagnostic gold standards for *N. gonorrhoeae* detection, owing to improved sensitivity, rapidity, and automation (Golparian et al., 2015; Perera et al., 2017). Nevertheless, NAATs are often inaccessible and unaffordable in resource-constrained settings due to expensive thermo-cycling instrumentation and the requirement for highly trained personnel.

Multiple cross displacement amplification (MCDA) is an innovative nucleic acid isothermal amplification technique proposed as an attractive alternative to traditional PCR-related techniques (Wang et al., 2017). The strategy has potential applications as a point-of-care (POC) assay due to its rapidity, simplicity, and easy-to-use operations. Critically, it has been used to detect several pathogens such as SARS-CoV-2, hepatitis B virus, and *Streptococcus agalactiae* (Cheng et al., 2020; Li et al., 2020; Chen et al., 2021). The gold nanoparticle-based lateral flow biosensor (LFB) is a paper-based platform, which facilitates a low-cost rapid diagnosis; it is robust, visual, sensitive, specific, and has a low limit of detection (LoD) (Zeng et al., 2013; Urusov et al., 2019). Importantly, the technique can be used in clinical settings to detect specific biomarkers, including antibodies, antigens, and DNA; therefore it is considered a new and easy-to-use POC device.

**FIGURE 1:**
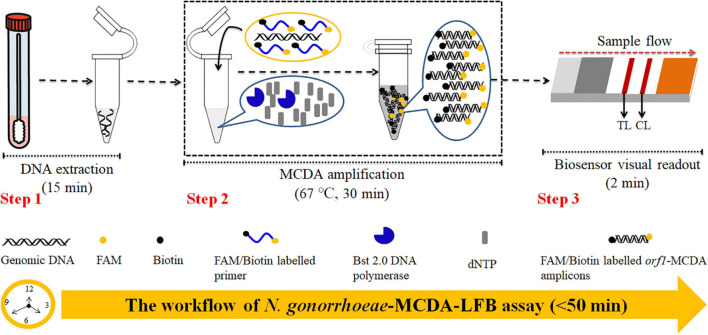
*Neisseria gonorrhoeae*-multiple cross displacement amplification-lateral flow biosensor (MCDA-LFB) workflow. *N. gonorrhoeae*-MCDA-LFB assay uses three closely linked steps: DNA extraction (step 1), multiple cross displacement amplification (MCDA) (step 2), and gold nanoparticle-based lateral flow biosensor (LFB) readout (step 3). The detection process takes approximately 50 min.

**FIGURE 2:**
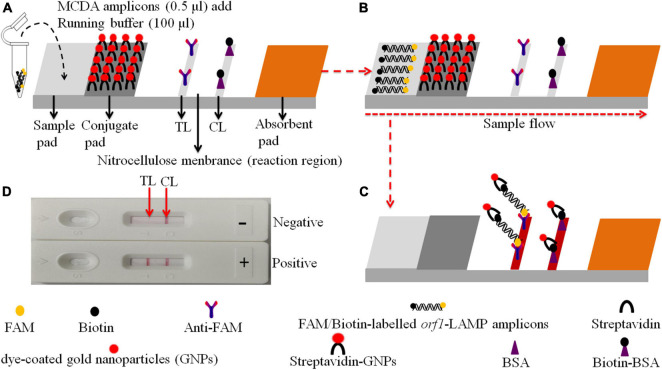
Schematic of the gold nanoparticle-based LFB for *N. gonorrhoeae*-MCDA product visualization. **(A)** MCDA products (0.5 μl) and running buffer (100 μl) were deposited on the sample pad. **(B)** The running buffer containing MCDA products moves along the LFB due to capillary action, while streptavidin dye-coated gold nanoparticles (streptavidin-DPNs) are rehydrated in the conjugate region. **(C)** For positive samples, FAM/biotin-labeled *orf1*-MCDA amplicons are captured by anti-carboxyfluorescein (FAM) at the test line (TL), and streptavidin-DPNs are captured by biotin-BSA at the control line (CL). For negative outcomes, only streptavidin-DPNs are captured by biotinylated bovine serum albumin (biotin-BSA) at the CL. **(D)** Interpretation of *N. gonorrhoeae*-MCDA-LFB assay results; positive – CL and TL appear on the LFB, and negative – only the CL appears on the LFB.

In our study, MCDA combined with gold nanoparticle-based LFB (MCDA-LFB) was constructed to detect the *N. gonorrhoeae orf1* gene (Chaudhry and Saluja, 2002; Edwards et al., 2014), which appeared to be uniquely present in *N. gonorrhoeae* as it showed no homology with other microbial genomes at GenBank by BLAST searches The assay principle and study workflow is outlined in Figures 1, 2, and we validated its feasibility using patient clinical samples. The assay was completed with high accuracy within 50 min. Therefore, the MCDA-LFB assay can be considered a valuable POC testing device for diagnosing gonorrhea, even in financially impoverished settings.

## Materials and Methods

### Reagents

Nucleic acid releasing agents were obtained from Sansure Biotech Inc. (Changsha, China). Thayer–Martin (TM) chocolate agar plates were purchased from Autobio Biotechnology Co., Ltd. (Zhengzhou, China). The colorimetric indicator, malachite green (MG) and universal isothermal amplification kits were obtained from HuiDeXin Bio-Technique (Tianjin, China). Biotinylated bovine serum albumin (Biotin-BSA) and rabbit anti-fluorescein antibody (anti-FAM) were purchased from Abcam Co., Ltd. (Shanghai, China). Streptavidin dye-coated gold nanoparticles (Crimson red) were obtained from Bangs Laboratories, Inc. (IN, United States). Gold nanoparticle-based LFB materials, including sample pad, conjugate pad, absorbent pad, nitrocellulose membranes, and backing cards were manufactured by HuiDeXing Biotech Co., Ltd. (Tianjing, China) according to our design specifications. Gonococcus commercial PCR diagnosis kits were obtained from DaAn Gene Co., Ltd. (Guangzhou, China).

### Clinical Sample Preparation

We collected 116 genital secretion samples from suspected *N. gonorrhoeae*-infected patients at the Hangzhou Women’s Hospital between August 2020 and April 2021. Two samples were collected from patients using sterile swabs. One sample was immediately inoculated on Thayer-Martin (TM) chocolate agar plates at 37°C, 80% humidity, pH 6.8–7.5, and in a 5% CO2-enriched atmosphere for 2–3 d. The remaining sample was for nucleic acid extraction. *N. gonorrhoeae* genomic DNA was obtained using nucleic acid releasing agents in accordance with manufacturer’s instructions. In brief, the collected genital secretion samples (500 μl) were centrifuged at 12000 rpm at 4°C for 5 min. The pellet was suspended in 50 μl nucleic acid releasing agents, and incubated at room temperature (25°C) for 10 min. Genomic DNA concentrations were measured using a Nano Drop ND-2000 (Thermo, United States) at A260/280 nm. The corresponding genome copy number was calculated from the weight of the *N. gonorrhoeae* genome, One *N. gonorrhoeae* genome is 2.45 fg (2.2 × 10^6^ bp (Dempsey et al., 1991) × 665 Da/bp × 1.67 × 10^−24^ g/Da) (Edwards et al., 2014).

### Gold Nanoparticle-Based Lateral Flow Biosensor Preparations

The gold nanoparticle-based LFB design is shown in Figure 2. Briefly, the LFB was composed of the following sections: sample pad, conjugate pad, nitrocellulose membrane with immobilized anti-FAM and biotin-BSA, and an absorbent pad. Dye streptavidin-coated gold nanoparticles were deposited on the conjugate pad. Biotin-BSA and anti-FAM were dispensed onto the nitrocellulose membrane to act as the control line (CL) and test line (TL) (*N. gonorrhoeae*), respectively. Each band was separated by 5 mm. The biosensor components were affixed to a backing card.

### *Neisseria gonorrhoeae*-Multiple Cross Displacement Amplification Primer Design

Based on MCDA reaction mechanisms, five pairs of primers based on *orf1* (GenBank Accession No. M84113) were designed using primer Explorer V5 and PRIMER PREMIER 5.0 software (Eiken Chemical, Japan). A set of *N. gonorrhoeae*-MCDA primers, including displacement primers (F1 and F2), cross primers (CP1 and CP2), and amplification primers (D1, D2, C1, C2, R1, and R2), was also generated (Table 1). *N. gonorrhoeae*-MCDA primer specificity was verified using the BLAST analysis tool. In addition, Oligo Analyzer online software (V3.1; Integrated DNA Technologies, Coralville, IA, United States) was used for primer secondary structure and dimer investigations. MCDA primer sequences are shown (Table 1). All primers were synthesized and purified at TsingKe Biotech Co., Ltd. (Beijing, China) using high performance liquid chromatography purification grade.

**TABLE 1:** *Neisseria gonorrhoeae*-multiple cross displacement amplification (MCDA) primers used in this study.

**Primer name**	**Sequence and modifications**	**Length**	**Gene**
F1	5′-ACGTCCACCAATCCATTTGG-3′	20 nt	*orf1*
F2	5′-GATGGAAGCGGAACGGTT-3′	18 nt	
CP1	5′-TGTAGTAGAGCGCGGTATCGGAACGGTCAAAACCTGTTCGC-3′	41 mer	
CP2	5′-ACCAACTCCTACAAACGCCTCGTTGGCGGAATAGGCCAATT-3′	41 mer	
C1	5′-TGTAGTAGAGCGCGGTATCGGA-3′	22 nt	
C1*	5′-Biotin-TGTAGTAGAGCGCGGTATCGGA-3′	22 nt	
C2	5′-ACCAACTCCTACAAACGCCTCG-3′	22 nt	
D1	5′-AAACCGGCATAGCCGTCG-3′	18 nt	
D1*	5′-FAM-AAACCGGCATAGCCGTCG-3′	18 nt	
D2	5′-ACTTTGAAGCACCGACC-3′	17 nt	
R1	5′-GCTTTGGCGTGTTTGAT-3′	17 nt	
R2	5′-TGAACGCGATTACCAAT-3′	17 nt	

C1*, 5′-labeled with biotin when used for MCDA-LFB detection; D1*, 5′-labeled with FAM when used for MCDA-LFB detection. FAM, 6-carboxy-fluorescein; nt, nucleotide; mer, monomeric unit.

### Multiple Cross Displacement Amplification and Detection

Multiple cross displacement amplification was performed using an isothermal amplification kit (HuiDeXing Biotech Co., Ltd., Tianjing, China). Briefly, a one-step 25-μl reaction mixture contained 12.5-μl 2 × reaction buffer [40 mM Tris–HCl (pH 8.8), 40 mM KCl, 16 mM MgSO_4_, 20 mM (NH_4_)_2_SO_4_, 2 M betaine, and 0.2% Tween-20]; 1 μl (8 U) *Bst* 2.0 DNA polymerase (New England Biolabs, United States); 1 μl (10 U) AMV Reverse Transcriptase (only used for RNA templates); 0.4 μM F1 and F2 primers; 1.6 μM CP1 and CP2 primers; 1.2 μM of D1^∗^, D2, C1^∗^, C2, R1, and R2 primers; 2-μl nucleic acid template; and doubly distilled water (DW) to 25 μl. The reaction was performed in heat-block at constant temperature (optimization outlined later).

Monitoring techniques, including the colorimetric indicator, MG, real-time turbidity (LA-500), and an LFB were used to analyze MCDA products. For colorimetric analysis, colorless reactions changed to light green suggesting a positive amplification. A colorless reaction was observed in negative and blank controls (BCs). For the real-time turbidity method, turbidity >0.1 was regarded as a positive result. For LFB detection, the CL and TL reacted simultaneously, indicating positive results. For negative and blank outcomes, only the CL was observed on the biosensor.

### Optimization of *Neisseria gonorrhoeae*-Multiple Cross Displacement Amplication-Lateral Flow Biosensor Reaction Conditions

Temperature is critical for isothermal amplification. The amplification temperature was optimized, ranging from 63 to 70°C (with 1°C intervals) using *N. gonorrhoeae* genomic DNA (2.0 × 10^3^ copies/assay). MCDA amplicons were monitored using real-time turbidity (LA-500). Then, amplification times (20, 30, 40, and 50 min) of the assay were optimized. The *N. gonorrhoeae*-MCDA reaction was carried out with optimal amplification temperature, and the results were readout simultaneously with MG and LFB. Each test was conducted at least three times.

### Sensitivity and Specificity of the *Neisseria gonorrhoeae*-Multiple Cross Displacement Amplification-Lateral Flow Biosensor Assay

To test assay sensitivity, reference *N. gonorrhoeae* [American type culture collection (ATCC); 49926] genomic DNA was 10-fold serial diluted from 2.0 × 10^4^ to 2.0 × 10^–1^ copies. Then, *N. gonorrhoeae*-MCDA reactions were conducted under optimal conditions, and the results were analyzed using MG and LFB. The LoD of *N. gonorrhoeae*-MCDA was confirmed as the lowest dilution for which all three replicates were positive. Assay specificity was determined using nucleic acid (at least 2.0 × 10^4^/reaction) from various bacteria, viruses, and fungi (Table 2). DW was used as a BC. MCDA results were monitored using an LFB and each assay was performed in triplicate.

**TABLE 2:** Pathogens used in this study.

**No.**	**Pathogen**	**Source of pathogens^a^**	**No. of strains**	***N. gonorrhoeae*-MCDA result^b^**
1	*N. gonorrhoeae* (reference strain)	ATCC 49926	1	P
2	*N. gonorrhoeae* (clinical samples)	Hangzhou Women’s Hospital	8	P
3	*Neisseria meningitides*	Hangzhou Women’s Hospital	1	N
4	*Chlamydia trachomatis*	Hangzhou Women’s Hospital	1	N
5	Enteropathogenic *Escherichia coli*	GZCCL	1	N
6	*Haemophilus influenzae*	ATCC49247	1	N
7	*Pseudomonas aeruginosa*	2nd GZUTCM	1	N
8	*Candida glabrata*	2nd GZUTCM	1	N
9	*Staphylococcus aureus*	2nd GZUTCM	1	N
10	*Acinetobacter baumannii*	2nd GZUTCM	1	N
11	*Streptococcus pyogenes*	2nd GZUTCM	1	N
12	*Bordetella pertussis*	GZCCL	1	N
13	*Mycobacterium tuberculosis*	GZCDC	1	N
14	*Cryptococcus neoformans*	ATCC13690	1	N
15	*Leptospira interrogans*	GZCDC	1	N
16	*Klebsiella pneumoniae*	GZCCL	1	N
17	*Shigella flexneri*	Hangzhou Women’s Hospital	1	N
18	*Mycoplasma pneumonia*	Hangzhou Women’s Hospital	1	N
19	*Haemophilus parainfluenzae*	GZCCL	1	N
20	Human rhinovirus	Hangzhou Women’s Hospital	1	N
21	Respiratory syncytial virus type A	Hangzhou Women’s Hospital	1	N
22	Adenoviruses	Hangzhou Women’s Hospital	1	N

*^*a*^ATCC, American type culture collection; 2nd GZUTCM, the Second Affiliated Hospital, Guizhou University of Traditional Chinese Medicine; GZCCL, Guizhou Provincial Center for Clinical Laboratory; GZCDC, Guizhou Provincial Center for Disease Control and Prevention.^*b*^P, positive; N, negative.*

### Verification of *Neisseria gonorrhoeae*-Multiple Cross Displacement Amplification-Lateral Flow Biosensor Feasibility Using Clinical Samples

To confirm assay feasibility, the optimized detection system was verified using clinical samples. We collected 116 suspected *N. gonorrhoeae*-infection genital secretion samples from Hangzhou Women’s Hospital (Hangzhou, China). Samples were simultaneously assayed using cultivation, quantitative PCR (qPCR), and MCDA-LFB methods.

Culturing was conducted as above described. qPCR detection was performed using the Gonococcus nucleic acid assay kit (DaAn Gene Co., Ltd., China) (Cat. #DA-D053) and detection was performed using the Applied Biosystems^TM^ 7500 Real-Time PCR System (Life Technologies, Singapore). According to the instruction of the manufacturer, *N. gonorrhoeae* concentrations <500 copies were regarded as negative results The *N. gonorrhoeae*-MCDA-LFB operation was as described above. All assays were conducted at biosafety level 2 according to the WHO Laboratory Biosafety Manual, 3rd edition.

### Statistical Analysis

The χ^2^ test was used to compare sensitivity differences between cultivation, qPCR, and MCDA-LFB assays. The SPSS 23.0 software was used for statistical analyses, and *p* < 0.05 was considered statistically significant.

## Results

### Overview of the *Neisseria gonorrhoeae*-Multiple Cross Displacement Amplification-Lateral Flow Biosensor Detection System

The *N. gonorrhoeae*-MCDA-LFB system and workflow is shown in Figures 1, 2. Briefly, released *N. gonorrhoeae* genomic DNA was pre-amplified by MCDA at a constant temperature of 67°C for 30 min. We modified the C1 and D1 MCDA primers at the 5′-end with biotin and FAM for LFB detection. Amplicons were then labeled with biotin and FAM (Figures 1, 2). For positive samples, FAM/biotin-labeled *orf1*-MCDA amplicons were captured by anti-FAM at the TL, and streptavidin-DPNs were captured by biotin-BSA at the CL. For a negative outcome, only streptavidin-DPNs were captured by biotin-BSA at the CL (Figure 2).

### Demonstration of the *Neisseria gonorrhoeae*-Multiple Cross Displacement Amplification Assay

**FIGURE 3:**
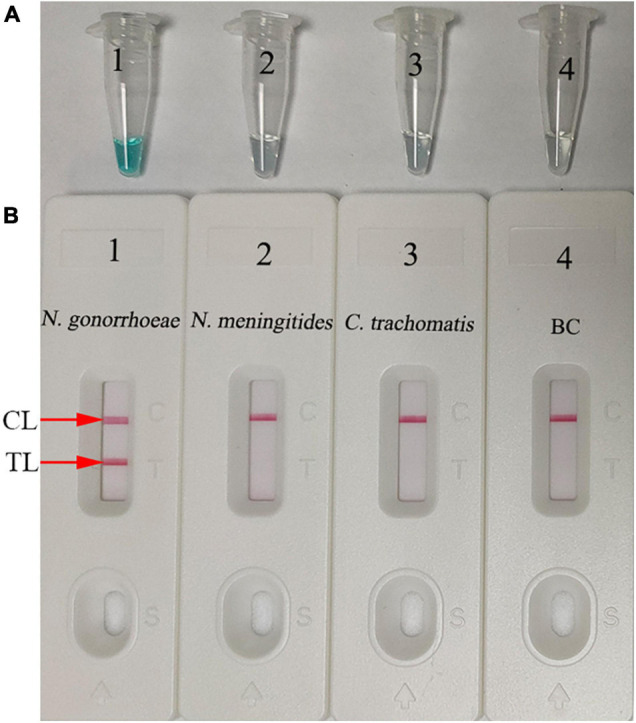
Determination and confirmation of *N. gonorrhoeae-*MCDA products. *N. gonorrhoeae*-MCDA products were monitored using a colorimetric indicator **(A)** and a gold nanoparticle-based biosensor **(B)**. Tube 1/Biosensor 1: positive result for the *N. gonorrhoeae* reference strain (ATCC 49926); Tube 2/Biosensor 2: negative result for *Neisseria meningitides*; Tube 3/Biosensor 3: negative result for *Chlamydia trachomatis*; Tube 4/Biosensor 4: blank control (distilled water). TL, test line; CL, control line.

To validate *N. gonorrhoeae*-MCDA primers, MCDA reaction mixtures were incubated on a heat-block at a constant temperature of 65°C for 1 h using nucleic acid from purified *N. gonorrhoeae* (ATCC 49926) cultures. MCDA amplicons were then analyzed using MG and LFB techniques. Our data showed that the color of the *N. gonorrhoeae*-MCDA tube went from colorlessness to bright green, while negative and BCs remained colorless (Figure 3A). Using LFB detection, TL and CL appeared simultaneously in the *N. gonorrhoeae*-MCDA reaction, while in negative and BCs, only the CL appeared (Figure 3B). These data suggested that the *N. gonorrhoeae*-MCDA primers for *orf1* detection were valid for ongoing assay development.

### Determining the Optimal Amplification Temperature of the *Neisseria gonorrhoeae*-Multiple Cross Displacement Amplification-Lateral Flow Biosensor Assay

**FIGURE 4:**
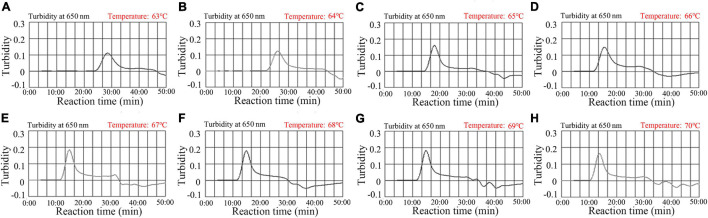
Temperature optimization for *N. gonorrhoeae-*MCDA reactions. *N. gonorrhoeae-*MCDA reactions were monitored by real-time turbidity measurements (LA-500). Turbidity >0.1 was considered positive, and the threshold value was 0.1. Eight kinetic graphs **(A–H)** were derived at various reaction temperatures; 63–70°C at 1°C intervals, with *N. gonorrhoeae* reference strain (ATCC 49926) target DNA at 2 × 10^3^ copies/reaction. The optimal MCDA reaction temperature was selected based on higher turbidity. The temperature, 67°C **(E)** showed robust amplification.

Reaction temperature is important for isothermal amplification. For the MCDA pre-amplification stage, temperatures from 63–70°C with 2.0 × 10^3^ copies/reaction of *N. gonorrhoeae* DNA template were investigated. Using real-time turbidity (LA-500) (Figure 4), the robust amplification of *N. gonorrhoeae*-MCDA was observed at 67–69°C. Therefore, 67°C was used as the optimal reaction temperature for subsequent studies.

### Sensitivity of the *Neisseria gonorrhoeae*-Multiple Cross Displacement Amplification-Lateral Flow Biosensor Assay

**FIGURE 5:**
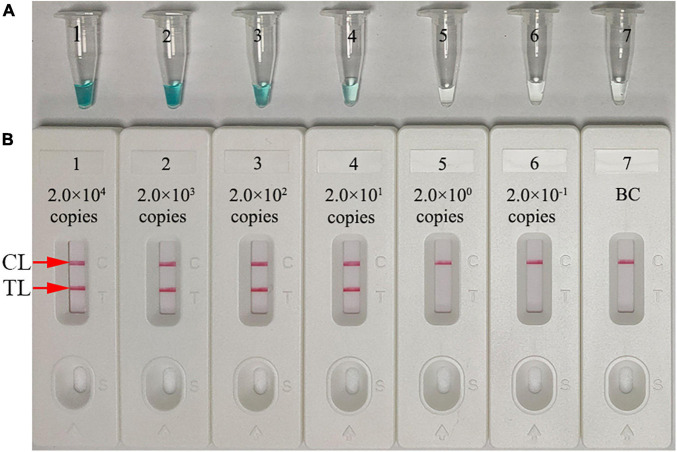
Assay sensitivity using diluted genomic DNA templates. Malachite green (MG) **(A)** and gold nanoparticle-based LFB **(B)** techniques were simultaneously used for reporting *N. gonorrhoeae-*MCDA results. Tubes A1–A7 (Biosensors B1–B7) contained 2.0 × 10^4^, 2.0 × 10^3^, 2.0 × 10^2^, 2.0 × 10^1^, 2.0 × 10^0^, and 2.0 × 10^–1^ DNA copies/reaction and blank control (distilled water). The limit of detection (LoD) of the *N. gonorrhoeae-*MCDA-LFB assay for *orf1* was 2.0 × 10^1^ DNA copies/reaction. CL, control line; TL, test line.

Assay sensitivity was tested using serial dilutions of *N. gonorrhoeae* DNA (from 2.0 × 10^4^ to 2.0 × 10^–1^ copies/test). The assay was conducted as described, and the results were analyzed using MG and LFB. The assay LoD was 20 copies/test (in a 25-μl reaction mixture) (Figures 5A,B).

### Optimal Amplification Times for the *Neisseria gonorrhoeae*-Multiple Cross Displacement Amplification-Lateral Flow Biosensor Assay

**FIGURE 6:**
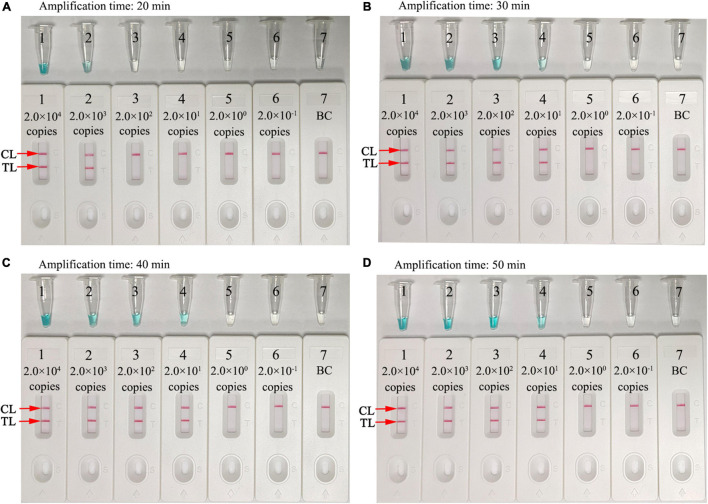
Optimization of assay reaction times. Four different amplification times **(A)**, 20 min; **(B)**, 30 min; **(C)**, 40 min; and **(D)**, 50 min were tested at 67°C. Tube/Biosensors 1, 2, 3, 4, 5, 6, and 7 represented *N. gonorrhoeae* (ATCC 49926) genomic DNA at 2.0 × 10^4^ to 2.0 × 10^–1^ copies/reaction and blank control (distilled water). Optimal sensitivity was indicated when the amplification lasted 30 min **(B)**. CL, control line; T, test line.

Amplification assay times of 20, 30, 40, and 50 min were investigated and optimized at 67°C. MCDA products were analyzed using MG and LFB. As shown in Figure 6, the LoD level of *N. gonorrhoeae* template (20 copies) was tested when the amplification lasted for 30, 40, and 50 min. Hence, a 30-min reaction time was recommended for assay at the MCDA pre-amplification stage.

### Specificity of the *Neisseria gonorrhoeae*-Multiple Cross Displacement Amplification-Lateral Flow Biosensor Assay

**FIGURE 7:**
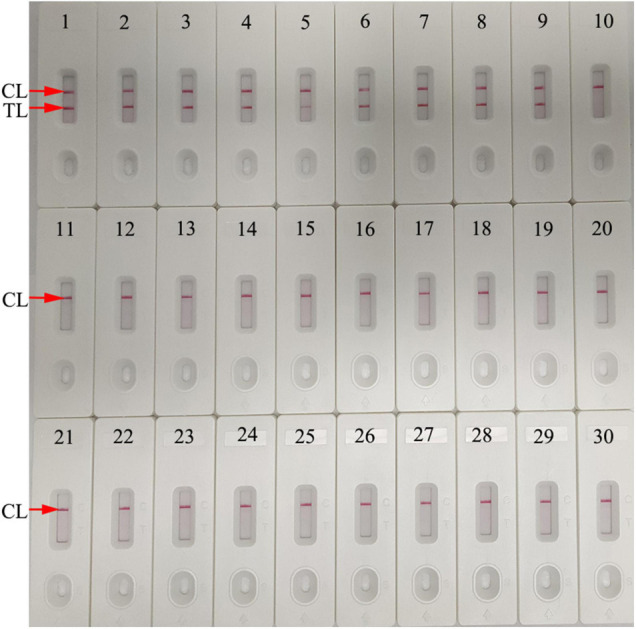
Assay specificity using different pathogens. The *N. gonorrhoeae-*MCDA-LFB assay was verified using different genomic RNA/DNA as templates. Biosensor 1, *N. gonorrhoeae* (ATCC 49926); Biosensors 2–9, *N. gonorrhoeae* (clinical samples); Biosensor 10, *Neisseria meningitides*; Biosensor 11, *Chlamydia trachomatis*; Biosensor 12, Enteropathogenic *Escherichia coli*; Biosensor 13, *Haemophilus influenzae*; Biosensor 14, *Pseudomonas aeruginosa*; Biosensor 15, *Candida glabrata*; Biosensor 16, *Staphylococcus aureus*; Biosensor 17, *Acinetobacter baumannii*; Biosensor 18, *Streptococcus pyogenes*; Biosensor 19, *Bordetella pertussis*; Biosensor 20, *Mycobacterium tuberculosis*; Biosensor 21, *Cryptococcus neoformans*; Biosensor 22, *Leptospira interrogans*; Biosensor 23, *Klebsiella pneumoniae*; Biosensor 24, *Shigella flexneri*; Biosensor 25, *Mycoplasma pneumonia*; Biosensor 26, *Haemophilus parainfluenzae*; Biosensor 27, human rhinovirus; Biosensor 28, respiratory syncytial virus type A; Biosensor 29, adenoviruses; Biosensor 30, blank control (distilled water). CL, control line; TL, test line.

Assay specificity was evaluated and verified using an *N. gonorrhoeae* reference strain (ATCC 49926), eight *N. gonorrhoeae*-positive clinical samples (confirmed with cultivation), and 20 non-*N. gonorrhoeae* samples (bacteria, viruses, and fungi) (Table 2). Optimal assay reaction conditions were implemented as outlined, and the results were read using an LFB. Positive results were only observed from *N. gonorrhoeae* templates, while other pathogens and BCs were negative (Figure 7). No cross-reactions were observed. Combined, these data suggested that the *N. gonorrhoeae*-MCDA-LFB assay was highly selective for *orf1*.

### Confirming *Neisseria gonorrhoeae*-Multiple Cross Displacement Amplification-Lateral Flow Biosensor Assay Feasibility Using Clinical Specimens

We used 116 suspected *N. gonorrhoeae*-infection samples for analysis, and then, all of the samples were detected simultaneously with culture-biotechnical, qPCR, and MCDA-LFB. Our data showed that 47 of 116 samples were *N. gonorrhoeae*-positive using the *N. gonorrhoeae*-MCDA-LFB assay. Cultivation assay data were also consistent with this result. For qPCR, only 45 samples were confirmed as positive (Table 3 and Supplementary Table 1). These results suggested our *N. gonorrhoeae*-MCDA-LFB assay functioned as an advanced gonorrhea diagnostic tool for clinical specimens.

**TABLE 3:** Conventional cultivation, quantitative polymerase chain reaction (qPCR), and multiple cross displacement amplification-lateral flow biosensor (MCDA-LFB) comparisons for *N. gonorrhoeae* testing in clinical samples.

**Detection assay**	**Results**	**Gold standard method (cultivation)**		**True positive rate (%)**	**True negative rate (%)**
		**+**	**−**		
qPCR	+	45	0	95.7^a^	100
	−	2	69		
MCDA-LFB	+	47	0	100	100
	−	0	69		

*^*a*^Statistically significant (*p* < 0.05) when compared with MCDA-LFB.*

## Discussion

The Gram-negative diplococcus, *N. gonorrhoeae* causes sexually transmitted gonorrhea and is a major global public health concern (Comunian-Carrasco et al., 2018; Vallely et al., 2021). The overwhelming majority of gonococcal infections are in less-developed regions, likely owing to a lack of well-functioning public health infrastructure and poor availability of laboratory diagnostics (Kirkcaldy et al., 2019). Therefore, a reliable, cost-saving, rapid, sensitive, and easy-to-use assay is critical for addressing the ever-increasing gonorrhea transmission rates. In this study, a novel *N. gonorrhoeae*-MCDA-LFB assay, which integrated gold nanoparticle-based LFB with MCDA was devised and successfully applied to the rapid and visual identification of *N. gonorrhoeae* in clinical specimens.

The ideal laboratory-based diagnostic method should be accurate, sensitive, specific, rapid, cheap, and easy-to-use. Traditionally, cultivation was considered the gold standard for *N. gonorrhoeae* detection; however, the bacteria are fastidious and require specific culture conditions, otherwise false negative results may be generated (Meyer and Buder, 2020; Visser et al., 2020). Furthermore, culturing is labor intensive and time consuming (approximately 2 days). Compared with cultivation, direct microscopy is relatively simple and rapid, but is not recommended as a diagnostic method owing to low sensitivity (Thorley and Radcliffe, 2015; Meyer and Buder, 2020). NAATs are more sensitive and specific and are considered primary diagnostic methods for *N. gonorrhoeae* detection (Perera et al., 2017; Dona et al., 2018). However, in underdeveloped regions, their use is confined due to the requirement for trained operators and expensive thermal cyclers. Here, we developed an *N. gonorrhoeae*-MCDA-LFB technique requiring simple instruments, a heating block, water bath, or even a thermos cup that can hold 67°C for 30 min. Moreover, amplification products were objectively visualized using an LFB. The detection process, including genomic DNA extraction (∼15 min), MCDA amplification (30 min), and result readout (∼2 min) can be accomplished within 50 min. In the current study, the method of template preparation (nucleic acid-releasing agents) contributed to the rapidity of the assay and simplified the detection process. It is a relatively crude extraction technique, and more investigation is needed to empirically confirm this method.

To improve *N. gonorrhoeae*-MCDA-LFB assay sensitivity, MCDA was used to pre-amplify *orf1*. MCDA is a novel isothermal amplification method first devised by Wang et al. (2015) and is more sensitive than PCR and loop-mediated isothermal amplification methods (Wang et al., 2015; Zhu et al., 2021). The isothermal amplification of specific DNA sequences is performed using a set of 10 primers spanning 10 distinct regions of the target fragment; displacement primers (F1 and F2), cross primers (CP1 and CP2), and amplification primers (D1, D2, C1, C2, R1, and R2). The amplification procedure requires a *Bst* DNA polymerase with strand displacement capability at a single temperature (between 60°C and 68nd (Wang et al., 2015). In our study, a set of *N. gonorrhoeae*-MCDA primers were specifically designed to identify 10 regions in *orf1*. *N. gonorrhoeae*-MCDA-LFB assay specificity was verified using *N. gonorrhoeae* strains and other microbes. Positive results were only identified from gonococci isolates, and no cross reactions were observed with non-*N. gonorrhoeae* microbes (Table 2 and Figure 7). Hence, the *N. gonorrhoeae*-MCDA-LFB assay was highly specific for *N. gonorrhoeae* identification. Similarly, the assay detected as low as 20 *N. gonorrhoeae* copies/test. To identify *N. gonorrhoeae* in clinical samples, our assay displayed higher sensitivity than qPCR (Table 3 and Supplementary Table 1) and correctly diagnosed 100% (47/47) of *N. gonorrhoeae* samples identified by cultivation.

In this study, a gold nanoparticle-based LFB was used to detect *N. gonorrhoeae*-MCDA *orf1* amplicons. This paper-based platform is widely used in clinical settings due to its high selectivity, low LoD, low sample volume, low cost, robustness, rapidity, and user-friendly format (Quesada-González and Merkoçi, 2015; Koczula and Gallotta, 2016; Urusov et al., 2019; Wang et al., 2021). The LFB rapidly and visually detected *N. gonorrhoeae*-MCDA products for labeling with BSA-biotin and anti-FAM on LFB strips. Positive results were indicated by two crimson red bands on the LFB, CL, and TL. For negative outcomes, only the CL was indicated on the biosensor. Although real-time turbidity and MG generated *N. gonorrhoeae*-MCDA results, the former is costly and requires expensive equipment, while the latter is ambiguous when MCDA amplicon concentrations were low (Figure 5). The cost of each LFB in this study was calculated at approximately $2 US dollars (USD). Therefore, the total cost of each *N. gonorrhoeae*-MCDA-LFB test, including genomic DNA preparation (∼$1 USD), MCDA amplification (∼$3.5 USD), and LFB detection (∼$2 USD) was estimated at $6.5 USD. Isothermal amplification methods, including loop-mediated isothermal amplification (LAMP) and cross-priming amplification (CPA), have also been used to test *N. gonorrhoeae*. Edwards et al. (2014) utilized the LAMP assay for testing *N. gonorrhoeae* with a minimum of 20 copies per reaction. Yu et al. (2016) used CPA to test *N. gonorrhoeae* and *Chlamydia trachomatis* with the detection limits of 65 and 45 copies per reaction, respectively. However, these methods must rely on agarose gel electrophoresis and colorimetric indicator. In this study, we first combined MCDA amplification with LFB for diagnosis of *N. gonorrhoeae*, which is more convenience and rapid than that of methods.

The *N. gonorrhoeae*-MCDA-LFB also has some limitations. First, the molecular assays identify nucleic acid sequences specific for the pathogen. However, a positive result may indicate either an infectious or non-infectious agent. Second, the LFB detection must be taken off the lid of MCDA amplification tube. Also, there is a risk of contamination with post-reaction processing of MCDA amplicons. To control this, spraying a 10–15% sodium hypochlorite solution and 70% ethanol after LFB assay completion is effective in avoiding DNA contamination in laboratory conditions. We observed no cross contamination with non-*N. gonorrhoeae* isolates. It is indicated that the false positive rate has been effectively controlled in our laboratory.

## Conclusion

We successfully integrated an LFB platform with MCDA pre-amplification technology to devise a novel *N. gonorrhoeae*-MCDA-LFB assay for the sensitive, specific, rapid, cheap, and visual diagnosis of *N. gonorrhoeae* in clinical specimens. Our assay detected 20 genomic DNA copies/test and exhibited no cross reaction with other microbes. The detection process was approximately 50 min and did not require expensive apparatus. Hence, our assay shows great potential as a POC test for the identification of *N. gonorrhoeae* in clinical settings, especially in resource-limited regions.

## Data Availability Statement

The original contributions presented in the study are included in the article/Supplementary Material, further inquiries can be directed to the corresponding authors.

## Ethics Statement

The study was approved by the Human Ethics Committee of Hangzhou Women’s Hospital [Approval No. (2021)-K (2)-8] and complied with the Declaration of Helsinki. Before clinical samples/isolates were obtained, all personal patient identifiers were removed. Patient informed consent was waived by the Committee.

## Author Contributions

XC, LH, and SD designed and conceived the study and wrote and revised the manuscript. XC, LH, and YT participated in the primer design. QZ, YT, XT, and SD collected the clinical samples. XC, YT, QZ, and SD performed all the laboratory work. XC and QZ performed the statistical analyses. All authors read and approved the final manuscript.

## Conflict of Interest

The authors declare that the research was conducted in the absence of any commercial or financial relationships that could be construed as a potential conflict of interest.

## Publisher’s Note

All claims expressed in this article are solely those of the authors and do not necessarily represent those of their affiliated organizations, or those of the publisher, the editors and the reviewers. Any product that may be evaluated in this article, or claim that may be made by its manufacturer, is not guaranteed or endorsed by the publisher.

## Abbreviations

WHO: World Health Organization
MCDA: multiple cross displacement amplification
LFB: gold nanoparticle-based lateral flow biosensor
PCR: polymerase chain reaction
LoD: limit of detection
ATCC: American type culture collection
MG: malachite green
2nd GZUTCM: Second Affiliated Hospital, Guizhou University of Traditional Chinese Medicine
GZCCL: Guizhou Provincial Center for Clinical Laboratory
FAM: carboxyfluorescein
nt: nucleotide
mer: monomeric unit
TL: test line
CL: control line
BC: blank control
NC: negative control
DW: distilled water.
