# Supplementary material for: A Nanoparticle-Based Biosensor Combined With Multiple Cross Displacement Amplification for the Rapid and Visual Diagnosis of Neisseria gonorrhoeae in Clinical Application
Source: Front Microbiol. 2021 Oct 14;12:747140. doi: 10.3389/fmicb.2021.747140 (PMC8551913; doi:10.3389/fmicb.2021.747140)
Supplement: Supplementary file 1 [file Table_1.docx]

**Supplementary Materials**

**Nanoparticle-based biosensor combined with multiple cross displacement amplification for rapid and visual diagnosis of *Neisseria gonorrhoeae*in clinical application**

Xu Chen^1,2,3Δ*^, Liming Huang^1Δ^,Qingxue Zhou^4^, Yan Tan^5^, Xuhong Tan^1^, Shilei Dong^6*^

1. The Second Clinical College, Guizhou University of Traditional Chinese Medicine, Guiyang, Guizhou, 550003, People’s Republic of China
2. Clinical Medical Laboratory of the Second Affiliated Hospital, Guizhou University of Traditional Chinese Medicine, Guiyang, Guizhou, 550003, People’s Republic of China
3. GuizhouProvincial Centre for Disease Controland Prevention, Guiyang, Guizhou, 550004, People’s Republic of China
4. Clinical Laboratory, Hangzhou Women’s Hospital, Hangzhou, Zhejiang 310008, People’s Republic of China
5. Guizhou Provincial Center for Clinical Laboratory, Guiyang, Guizhou, 550002, People’s Republic of China
6. Department of Clinical Laboratory, Zhejiang Hospital, Hangzhou, Zhejiang 310013, People’s Republic of China

ΔXu Chen and Liming Huang contributed equally to this article.

*Corresponding author:

Xu Chen, E-mail: [xuchen1220@126.com](mailto:xuchen1220@126.com)

Shilei Dong, E-mail: dsl166@126.com

**TableS1:** Comparison of Cultivation, qPCR, and MCDA-LFB Assays for Detection of *N. gonorrhoeae*Using Clinical Samples

| **Sample NO.** | **Cultivation result** | **qPCR result（copies）** | **MCDA-LFB** |
| --- | --- | --- | --- |
| Test 1 | + | 4.2×10^3^ | + |
| Test 2 | + | 8.×10^3^ | + |
| Test 3 | + | 1.×10^7^ | + |
| Test 4 | + | 9.×10^5^ | + |
| Test 5 | + | 5.×10^6^ | + |
| Test 6 | + | 5.8×10^4^ | + |
| Test 7 | + | 4.1×10^4^ | + |
| Test 8 | + | 8.3×10^5^ | + |
| Test 9 | + | 5.3×10^7^ | + |
| Test 10 | + | 8.5×10^4^ | + |
| Test 11 | + | 4.4×10^3^ | + |
| Test 12 | + | 7.61×10^6^ | + |
| Test 13 | + | 8.4×10^6^ | + |
| Test 14 | + | 2.1×10^4^ | + |
| Test 15 | + | 1.1×10^3^ | + |
| Test 16 | + | <500（—） | + |
| Test 17 | + | 9.3×10^5^ | + |
| Test 18 | + | 4.4×10^5^ | + |
| Test 19 | + | 7.1×10^4^ | + |
| Test 20 | + | 3.8×10^6^ | + |
| Test 21 | + | 4.1×10^4^ | + |
| Test 22 | + | 2.6×10^4^ | + |
| Test 23 | + | 5.9×10^2^ | + |
| Test 24 | + | 1.2×10^7^ | + |
| Test 25 | + | 2.1×10^5^ | + |
| Test 26 | + | 1.2×10^7^ | + |
| Test 27 | + | 2.6×10^4^ | + |
| Test 28 | + | 3.1×10^5^ | + |
| Test 29 | + | 4.5×10^6^ | + |
| Test 30 | + | 4.3×10^4^ | + |
| Test 31 | + | 2.6×10^6^ | + |
| Test 32 | + | 2.92×10^4^ | + |
| Test 33 | + | 1.7×10^6^ | + |
| Test 34 | + | 3.4×10^6^ | + |
| Test 35 | + | 2.6×10^3^ | + |
| Test 36 | + | 6.1×10^2^ | + |
| Test 37 | + | 1.0×10^6^ | + |
| Test 38 | + | 1.1×10^7^ | + |
| Test 39 | + | 6.3×10^5^ | + |
| Test 40 | + | 5.1×10^7^ | + |
| Test 41 | + | 4.2×10^5^ | + |
| Test 42 | + | 3.8×10^6^ | + |
| Test 43 | + | <500（—） | + |
| Test 44 | + | 1.8×10^6^ | + |
| Test 45 | + | 7.9×10^4^ | + |
| Test 46 | + | 9.6×10^2^ | + |
| Test 47 | + | 6.7×10^4^ | + |
| Test 48-116 | — | — | — |

**Notice:** The qPCRdiagnosis was carried out using commercial real-time TaqMan PCR Kit (DaAn Gene Co., Ltd. China). The concentrations of *N. gonorrhoeae* less than 500 copies will be considered as negative resultsaccording to the manufacturer’s instructions.

+, Positive; —, Negative
